# Leveraging cfDNA fragmentomic features for the early detection of colorectal cancer

**DOI:** 10.3389/fimmu.2026.1705156

**Published:** 2026-01-28

**Authors:** Lina Shan, Dengyong Xu, Jie Chen, Wenjia Liu, Ji Lin, Juhang Bao, Jianfei Huang, Hanqing Zhang, Hanchen Zhao, Wei Xue, Ziao Lin, Bingjun Bai

**Affiliations:** 1 Department of Colorectal Surgery, Sir Run Run Shaw Hospital, School of Medicine, Zhejiang University, Hangzhou, China; 2 Department of Operating Room, Shangyu Hospital of Traditional Chinese Medicine, Shaoxing, China; 3 OmixScience Laboratory, OmixScience Co., Ltd, Hangzhou, China; 4 Department of Gastrointestinal Surgery, The First Affiliated Hospital of Wenzhou Medical University, Wenzhou, China; 5 Department of Colorectal Surgery, The First Affiliated Hospital of Ningbo University, Ningbo, China; 6 Department of Colorectal Surgery, Shaoxing People’s Hospital, Shaoxing, China; 7 University of California, Davis, Davis, CA, United States; 8 The Second Affiliated Hospital, Nanjing Medical University, Nanjing, China; 9 OmixScience Research Institute, OmixScience Co., Ltd., Shenzhen, China

**Keywords:** *Alu* elements, cell-free DNA, colorectal cancer, early detection, machine learning

## Abstract

**Background:**

Early detection of colorectal cancer (CRC) is crucial for improving patient outcomes. Cell-free DNA (cfDNA) analysis has emerged as a promising non-invasive approach for cancer detection. This study aims to develop a machine learning algorithm leveraging cfDNA fragmentomic features to accurately detect CRC.

**Methods:**

573 individuals from Sir Run Run Shaw Hospital, two community healthcare centers and three additional medical centers, were collected between April 1, 2023, and December 12, 2025. Participants were divided into training, internal validation, and external validation cohorts. A variety of cfDNA fragmentomic features were analyzed and incorporated into machine learning models. The models were evaluated using 10-fold cross-validation and assessed for accuracy, sensitivity, specificity, and AUC values. We also performed differential analysis of key genomic features, such as *Alu* elements and long terminal repeats (LTRs), between benign and malignant CRC samples.

**Results:**

The machine learning algorithm demonstrated robust discriminative performance across all datasets using generalized linear modeling (GLM), achieving AUC values of 0.959 (training set), 0.979 (internal validation cohort), and 0.959 (external validation cohort). Notably, the model exhibited particularly strong classification accuracy for advanced-stage colorectal cancer (CRC). Comparative cfDNA profiling revealed distinct molecular signatures between benign and malignant samples: benign samples were characterized by elevated frequencies of *Alu* elements and long terminal repeats (LTRs), whereas malignant samples showed distinct end motif profiles, characterized by the significant enrichment of specific 4-mer end motifs. These findings suggest that these molecular features may serve as potential biomarkers for malignancy detection.

**Conclusion:**

This study demonstrates that cfDNA fragmentomic profiling, particularly differential patterns of *Alu* and LTR elements, effectively discriminates benign from malignant colorectal lesions. These findings validate the clinical utility of repetitive element analysis and provide a foundation for developing improved non-invasive CRC diagnostics through machine learning approaches incorporating genomic features.

## Introduction

Colorectal cancer (CRC) is one of the leading causes of cancer-related mortality worldwide, with early detection being critical for improving patient prognosis and survival rates (1–3). Traditional screening methods, such as colonoscopy and fecal occult blood tests, while effective, are often invasive, expensive, or have limited sensitivity, particularly in early-stage disease (4, 5). As a result, there is a growing need for non-invasive, highly sensitive methods that can detect CRC at an earlier stage, thereby enabling timely intervention and treatment (6–9).

Cell-free DNA (cfDNA) has emerged as a promising biomarker for non-invasive cancer detection. cfDNA (10, 11), which consists of small fragments of DNA released into the bloodstream from apoptotic and necrotic cells, carries genetic and epigenetic information that reflects the state of the tumor and the surrounding tissue environment (12, 13). Recent advances in sequencing technologies and computational algorithms have enabled the detailed analysis of cfDNA fragmentomic features (14, 15), such as end breakpoint motifs (16–18), end motifs (19, 20), *Alu* elements (21, 22), LTRs (23), RNA elements, and transposable elements (24, 25). These features have shown potential in differentiating between benign and malignant conditions, making them valuable candidates for early cancer detection (26, 27). Some studies have already made some attempts in the detection of CRC by using cfDNA (28–30). However, more data are needed to support the clinical value of cfDNA fragmentomic features, especially in the early detection.

In this study, we sought to develop a machine learning algorithm that leverages cfDNA fragmentomic features to accurately detect CRC. By integrating a comprehensive set of genomic features into predictive models, we aimed to enhance the sensitivity and specificity of CRC detection, particularly in the early stages of the disease. GLM, an advanced machine learning techniques was utilized to develop and validate the predictive models. The findings of this study contribute significantly to the expanding research on cfDNA as a non-invasive diagnostic tool, while also underscoring the potential of advanced computational algorithms in enhancing the early detection of colorectal cancer. Through this research, we aim to offer new insights into the molecular mechanisms driving colorectal cancer and to identify potential biomarkers that could facilitate early detection.

## Methods and materials

### Participant enrollment and plasma sample collection

This study included 308 participants used for model training, and 133 participants as internal cohort for further validation of model, and 132 participants as external cohort for further validation of model. All of the individuals were previously untreated, and their diagnoses were confirmed by histopathology. The study was approved by the Ethics Committee of Sir Run Run Shaw Hospital, School of Medicine, Zhejiang University (No. 2023-02-3), the First Affiliated Hospital of Wenzhou Medical University (Ethics Approval No. 2022-202), the First Affiliated Hospital of Ningbo University (No. 2025-D052-02), and Shaoxing People’s Hospital (No. 2024-026-01). It adhered to the Declaration of Helsinki. All patients provided written informed consent prior to sample collection.

Patients with CRC were recruited according to predefined clinical inclusion and exclusion criteria. Eligible participants were adults (≥18 years) with pathologically confirmed CRC who had not received prior anti-tumor treatment, including surgery, chemotherapy, radiotherapy, or targeted therapy, before blood collection. Patients with a history of other malignancies, inflammatory bowel disease, active infections, autoimmune disorders, or severe systemic diseases were excluded to minimize potential confounding effects on cfDNA profiles. Blood samples were collected prior to any therapeutic intervention. Demographic and clinical variables, including age and sex, were recorded at enrollment, and their potential confounding effects were assessed during model development and validation to ensure balanced representation and robust performance across different age groups and between sexes.

### cfDNA assays and WGS

Peripheral blood samples were collected during routine physical examinations for healthy volunteers and prior to surgery for CRC patients. The blood samples were drawn and placed into cfDNA preservation tubes (Ardent BioMed, Guangdong, China, Cat. # BY10240301). After collection, the fresh blood was subjected to an initial centrifugation at 1,600 × g for 10 minutes at 4 °C, allowing the plasma supernatant to be carefully separated via pipetting. This plasma was then centrifuged again at 16,000 × g for 10 minutes at 4°C to remove any residual debris. The resulting plasma supernatant was then carefully collected and stored at -80°C. The preserved plasma samples were subsequently transported on dry ice to the central laboratory at OmixScience Research Institute (Hangzhou, China).

cfDNA was extracted from a median volume of 1 mL of plasma using the VAMNE MagUltra circulating cell-free DNA isolation kit (Vazyme Biotech, Nanjing, China, Cat. # N913) according to the manufacturer’s protocol. The extracted cfDNA was then quantified using a Qubit 4.0 fluorometer (Thermo Fisher Scientific, Lenexa, KS, USA) to ensure accurate measurement. Following quantification, the cfDNA was stored at -80 °C until further analysis. For library preparation, 5–20 ng of cfDNA was utilized with the VAHTS Universal DNA library prep kit for Illumina V3 (Vazyme Biotech, Nanjing, China, Cat. # N610) and barcoded using the VAHTS multiplex oligos set 5 for Illumina (Vazyme Biotech, Nanjing, China, Cat. # N322). The quality and integrity of the prepared libraries were assessed using the Agilent 2100 Bioanalyzer (Agilent Technologies, Santa Clara, CA, USA) to ensure they met the required standards for optimal fragment size distribution. Sequencing was performed on the NovaSeq X Plus platform (Illumina, San Diego, CA, USA) with paired-end 150bp reads, achieving an average coverage depth of 2× for the cfDNA samples.

Quality control of the raw whole-genome sequencing (WGS) data was conducted using FastQC (version 0.12.1, https://www.bioinformatics.babraham.ac.uk/projects/fastqc) to assess the overall quality. Low-quality reads and adapter sequences were removed and trimmed using Cutadapt (version 4.5, https://github.com/marcelm/cutadapt) and Ktrim (version 1.4.1, https://github.com/hellosunking/Ktrim). The filtered reads were then aligned to the human reference genome (GRCh37) using BWA-MEM (version 0.7.17, https://github.com/lh3/bwa) with default settings, ensuring only high-quality reads with a Phred score above the threshold were retained. Further analysis was restricted to uniquely mapped reads that were free of PCR duplicates. These mapped reads were subsequently sorted and indexed using Samtools (version 1.9, https://github.com/samtools/samtools), with duplicates identified and eliminated through Picard (version 2.18.29, https://github.com/broadinstitute/picard). The distribution of fragment sizes and relevant genomic features were inferred based on the mapped read pairs’ coordinates, following established protocols using Picard. To identify large-scale epigenetic variations in cfDNA fragmentation across the genome, which can be detected with low-coverage WGS, the hg19 reference genome was segmented into non-overlapping 5 Mb bins. Bins with an average GC content below 0.3 or average mappability below 0.9 were excluded from the analysis, leaving 473 bins that span approximately 2.4 Gb of the genome for further study.

### Fragmentomic feature identification

We analyzed the occurrence rates of 4 bp and 6 bp end motif patterns (16), ensuring that the cumulative frequency of all identified motifs in each category sums to one, providing comprehensive coverage. For the 6 bp end breakpoint motifs (18, 31), we carefully examined the 3 bp genomic DNA sequences both upstream and downstream of cfDNA 5’ end breakpoints. This detailed analysis allowed us to evaluate the prevalence of various 6 bp motifs across the entire human genome. To maintain consistency, the total frequency of all 6 bp breakpoint motifs was normalized to one. For subsequent analyses, we organized the motif features into an I × J matrix, where each row corresponds to a participant labeled with their sample ID. This matrix structure facilitates efficient interpretation and comparison of motif patterns across the cohort, enabling deeper insights into the observed cfDNA motif distributions.

For the analysis of regional fragment-size distribution, the genome was divided into 100 kb bins using Deeptools (version 3.1.2, https://github.com/deeptools/deepTools). Within each bin, the counts of short fragments (S, 100–150 bp) and long fragments (L, 151–220 bp) were calculated. The 100 kb bin size was chosen to balance between genomic resolution and achieving a sufficient number of reads per bin, targeting around 25,000 expected reads per bin to ensure an accurate estimation of the S/L ratio. To maintain data accuracy, regions that overlap with the ENCODE blacklist or the hg19 gap track from UCSC Genome Browser were excluded from the analysis, as these regions typically have poor mappability. By focusing on fewer but higher-quality bins, we enhanced the reliability of our fragment-size distribution analysis.

### Analysis of cfDNA using E-index/N-index ichorCNA

Plasma cfDNA was subjected to low-coverage whole-genome sequencing. Quantitative fragmentation indices including the N-index and E-index for DNA end characterization were calculated to assess end motif diversity and the evenness of fragment size distribution (32–34), respectively. In parallel, the same sequencing data were analyzed using the bioinformatics tool ichorCNA for genome-wide copy number alteration profiling and estimation of tumor fraction (32). All features were derived from the same batch of quality-controlled cfDNA sequencing data.

### Analysis of cfDNA using LIQUORICE and Griffin

For the analysis of regional fragment coverage, bamCoverage from deeptools (version 3.1.2) was initially used to calculate genome-wide read coverage for each cfDNA sample. To further analyze coverage data at specific regions of interest, such as cancer-associated regions of open chromatin, we employed the LIQUORICE tool (version 0.5.4, https://github.com/epigen/LIQUORICE). LIQUORICE performs a series of operations, including binning regions of interest, correcting for copy number alterations (CNA) biases, and calculating position-weight vectors to account for GC content, di-/trinucleotide composition, and mappability biases (35). Machine learning models, particularly random forests, were trained on these bias factors to predict and adjust coverage values. Subsequently, model-based fitting was applied to quantify coverage profiles across different levels of gene-regulatory regions. Gaussian functions were fitted to the aggregated and bias-corrected coverage profiles, with constraints based on biological parameters such as signal width and genomic location. The fitted parameters were then used to quantify dip strength and shape, providing insights into nucleosome occupancy and dynamics. This comprehensive approach allows for precise analysis of fragment coverage in regions of interest, taking into account various biases and regulatory factors.

We utilized the Griffin nucleosome profiling pipeline (version 0.2.0, https://github.com/adoebley/Griffin) to investigate nucleosome positioning around specific genomic sites (36, 37). The pipeline processes BAM files alongside GC bias information and a list of target sites. Reads within a predefined window around each target site are extracted, excluding those that do not pass quality control. After filtering read pairs based on fragment length, GC bias is calculated for each fragment, assigning a weight of 1 to each fragment and identifying its midpoint. A coverage profile is generated by summing the weighted fragment midpoints in 15 bp bins, with problematic and high-coverage bins excluded. The coverage profiles are then smoothed using a Savitzky-Golay filter and normalized to ensure consistent comparison across different samples. From each coverage profile, three key features are extracted: the coverage value at the target site, mean coverage within ±1,000 bp of the site, and nucleosome peak amplitude determined using Fast Fourier Transform within a specified window. GC normalization is performed to correct for differences in GC content between samples. This involves generating GC bias metrics, constructing averaged GC profiles using generalized additive models, and normalizing mutation counts according to GC content. Overall, this pipeline supports detailed nucleosome profiling, allowing for in-depth analysis of nucleosome positioning and GC bias at genomic sites of interest.

### *Alu* repeats analysis of cfDNA

We grouped all elements from the gene and pseudogene families, specifically transfer RNA (tRNA), signal recognition particle RNA (srpRNA), small nuclear RNA (snRNA), small cytoplasmic RNA (scRNA), and ribosomal RNA (rRNA)—under the category of RNA elements. Similarly, we classified elements from the DNA, retroposon, and RC (Rolling Circle) families as transposable elements. This classification left us with six major categories for analysis: LINE, SINE, LTR, satellites, transposable elements, and RNA elements. For our cfDNA feature analysis and validation, we focused on key elements such as *Alu* elements (22), long terminal repeats (LTRs) (23), RNA elements (21, 25), and transposable elements (23). By systematically organizing these components, we ensured a comprehensive and detailed approach to understanding cfDNA characteristics, which are crucial for elucidating their roles in colorectal cancer.

### Machine learning algorithm model

For tumor detection and classification, we conducted a comparative evaluation of two machine learning architectures: a GLM and a deep neural network (DNN). We constructed and selected the predictive model by systematically evaluating a wide range of cfDNA fragmentomic features in combination with machine learning approaches. The extracted features included end motifs (4 bp and 6 bp), breakpoint motifs (6 bp), E-index and N-index, CNA features derived from ichorCNA, Griffin-based features, LIQUORICE-based features, *Alu* elements, LTRs, RNA elements, and transposable elements. These genomic features have been extensively applied in distinguishing benign samples from malignant tumors. Using these features as inputs, both models were trained using motif feature frequencies derived from benign and malignant CRC samples in the training cohort to generate cancer score prediction models for individual samples. Cancer scores ranged from 0 to 1, with higher values indicating a greater likelihood of cancer presence. Model training and evaluation were performed using a rigorous bootstrapping strategy combined with 10-fold cross-validation, repeated 200 times. After finalizing model parameters based exclusively on the training cohort, cancer scores were calculated for each sample in the internal and external validation datasets, which were entirely independent of model training and parameter tuning to ensure an unbiased assessment of generalizability. Model performance was evaluated by comparing the area under the receiver operating characteristic curve (AUC), as well as sensitivity and specificity at predefined specificity thresholds across validation cohorts. Although the DNN exhibited strong performance even without parameter optimization, the GLM consistently outperformed the DNN, achieving higher AUCs in both internal and external validation sets and demonstrating superior sensitivity across all evaluated specificity thresholds. Based on these results, the GLM was selected as the final locked model for subsequent analyses. Relevant supporting literature for the selected features is cited accordingly.

### Statistics analysis

The code, software versions, and tabulated processed data used to generate the figures in this manuscript, along with details regarding the computing environment utilized for running the CRC_cfDNA pipeline, are available at the following GitHub repository (https://github.com/xueweireally/CRC_cfDNA). For statistical analyses involving two-group comparisons, Wilcoxon rank-sum tests were employed to calculate *P* values, as this robust non-parametric method is well-suited for assessing differences between groups. Additionally, the comparison of ROC curves was performed using the confusion matrix algorithm, which allows for a thorough evaluation of diagnostic test performance, ensuring accurate discrimination between positive and negative cases.

## Results

### Participant characteristics and disposition

**Figure 1:**
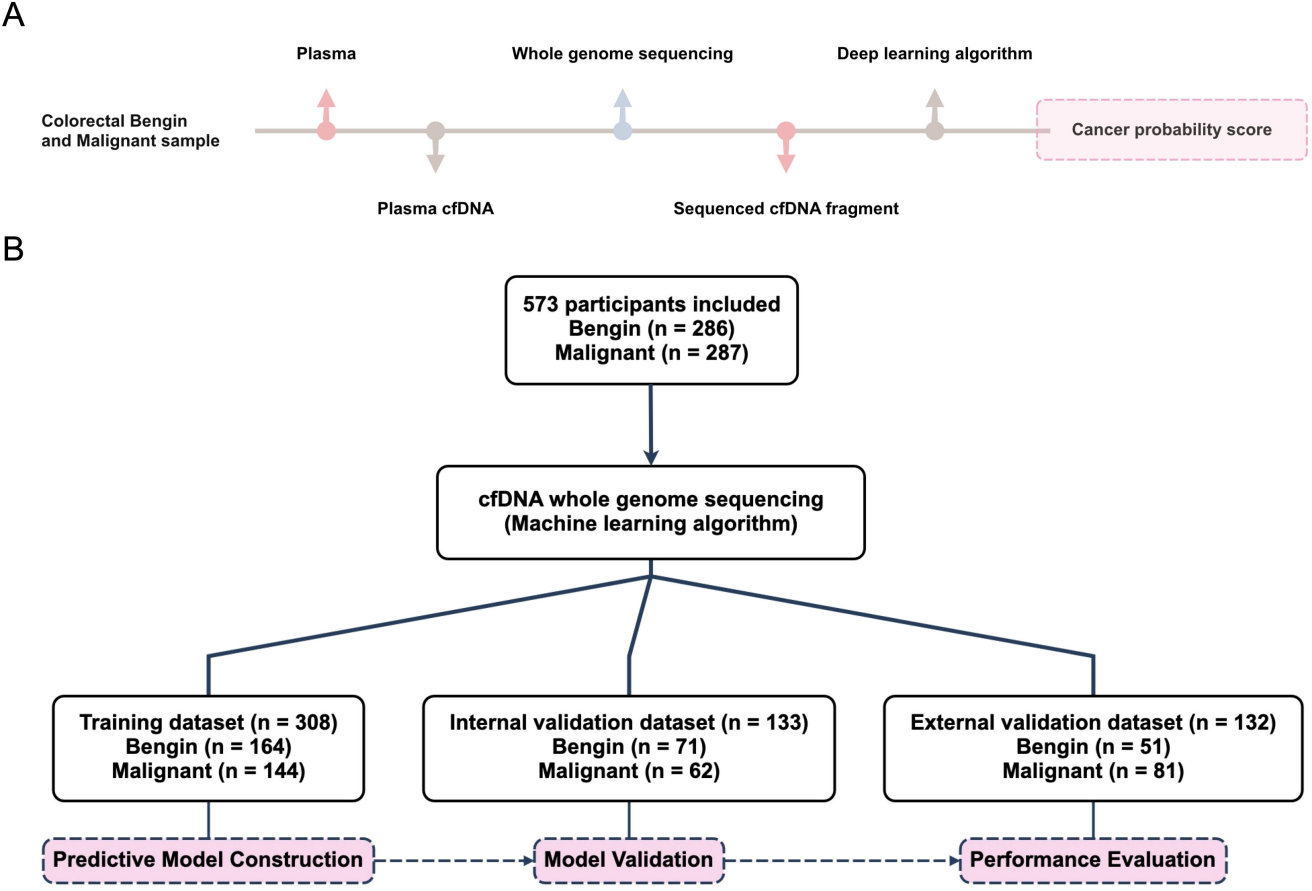
Workflow and participant inclusion for colorectal cancer cfDNA analysis. **(A)** The flowchart outlines the inclusion criteria and distribution of participants in the study. **(B)** The figure illustrates the step-by-step process of analyzing plasma cfDNA from colorectal cancer (CRC) and benign samples.

We included a total of 441 individuals at Sir Run Run Shaw Hospital, China, from October 1, 2023, to December 12, 2025. The participants were randomly divided into two groups in a 7:3 ratio into a training set (308 cases in total, 164 benign and 144 malignant) and an internal validation set (133 cases in total, 71 benign and 62 malignant). The training dataset consisted of samples was used for 10-fold cross-validation to develop the predictive CRC machine learning algorithm model. The internal validation cohort, which was used to assess the model’s performance. Additionally, we gathered samples from 132 participants from three medical centers, including the First Affiliated Hospital of Wenzhou Medical University, the First Affiliated Hospital of Ningbo University, and Shaoxing People’s Hospital, to create the external validation cohort, further ensuring the cancer predictive model’s accuracy and robustness (**Table 1** and **Supplementary Table S1**, **Figures 1A, B**). This comprehensive approach ensured a balanced and unbiased evaluation of the model’s effectiveness in detecting CRC.

**Table 1:** Demographic and clinical characteristics by pathology status.

Datasets	TDS (n=308)	IVD (n=133)	EVD (n=132)	Total (n=573)
Age (years)				
Mean (SD)	62.7 (12.1)	62.6 (13.1)	57.9 (14.0)	61.6 (12.9)
Median [Min, Max]	64.0 [30.0, 90.0]	64.0 [25.0, 90.0]	58.5 [23.0, 86.0]	63.0 [23.0, 90.0]
Age Group				
≤ 40	15 (4.9%)	9 (6.8%)	15 (11.4%)	39 (6.8%)
41-60	113 (36.7%)	45 (33.8%)	58 (43.9%)	216 (37.7%)
61-80	162 (52.6%)	67 (50.4%)	56 (42.4%)	285 (49.7%)
> 80	18 (5.8%)	12 (9.0%)	3 (2.3%)	33 (5.8%)
Gender				
Male	192 (62.3%)	83 (62.4%)	70 (53.0%)	345 (60.2%)
Female	116 (37.7%)	50 (37.6%)	62 (47.0%)	228 (39.8%)
Pathology status				
Benign	164 (53.2%)	71 (53.4%)	51 (38.6%)	286 (49.9%)
Malignant	144 (46.8%)	62 (46.6%)	81 (61.4%)	287 (50.1%)
Tumor stage				
I	31 (10.1%)	10 (7.5%)	16 (12.1%)	57 (9.9%)
II	27 (8.8%)	10 (7.5%)	25 (18.9%)	62 (10.8%)
III	78 (25.3%)	34 (25.6%)	25 (18.9%)	137 (23.9%)
IV	8 (2.6%)	8 (6.0%)	15 (11.4%)	31 (5.4%)
Missing	164 (53.2%)	71 (53.4%)	51 (38.6%)	286 (49.9%)

### Efficient predictive model for colorectal cancer detection

We constructed and selected the predictive model by systematically evaluating a wide range of cfDNA fragmentomic features in combination with machine learning approaches. The extracted features included end motifs (4 bp and 6 bp), breakpoint motifs (6 bp), E-index and N-index, copy number alteration (CNA) features derived from ichorCNA, Griffin-based features, LIQUORICE-based features, *Alu* elements, long terminal repeats (LTRs), RNA elements, and transposable elements. These genomic features have been extensively applied in distinguishing benign samples from malignant tumors. Using these features as inputs, we implemented two machine learning architectures, a GLM and a DNN, to compute a cancer probability score for differentiating benign and CRC samples.

Model performance was evaluated using both the internal validation cohort and an independent external validation cohort, with assessment based on the AUC and sensitivity at fixed specificity thresholds of 0.90, 0.95, and 0.99. The GLM consistently outperformed the DNN, achieving higher AUCs in both the internal validation set (GLM: 0.979 vs. DNN: 0.876) and the external validation set (GLM: 0.959 vs. DNN: 0.715), as well as superior sensitivity across all evaluated specificity thresholds (**Supplementary Table S2**). Accordingly, the GLM was selected as the final locked model for subsequent analyses.

### Performance of cancer predictive model

**Figure 2:**
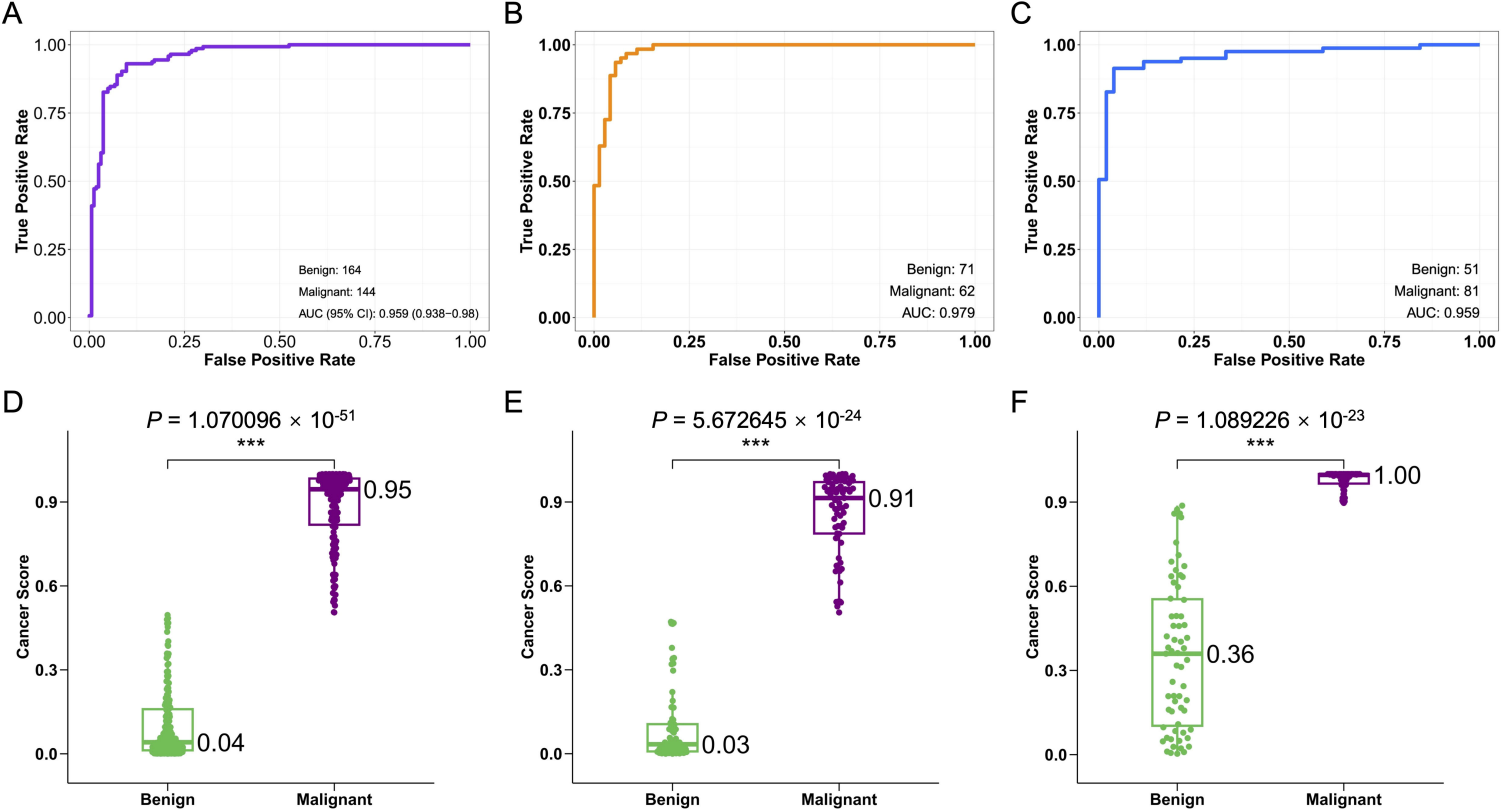
Performance of the CRC cfDNA machine learning algorithm model. **(A)** The ROC curves depict the performance of the CRC cfDNA machine learning algorithm model (GLM). **(B)** The ROC curve for the internal validation dataset shows the true positive rate against the false positive rate. **(C)** The ROC curve for the external validation dataset demonstrates the model’s performance. **(D)** Box plots of cancer scores for benign and malignant samples in the training dataset. **(E)** Box plots of cancer scores for the internal validation dataset. **(F)** Box plots of cancer scores for the external validation dataset. * indicates *P* < 0.05, ** indicates *P* < 0.01, and *** indicates *P* < 0.001, statistical analysis was performed using the Wilcoxon rank sum test.

The cancer predictive model demonstrated high accuracy in detecting CRC from plasma cfDNA samples using GLM model. In the training dataset, the model achieved an accuracy of 90.3%, with an AUC of 0.959, and exhibited strong sensitivity (0.889) and specificity (0.915) (**Figures 2A** and **S1A**). The internal validation dataset confirmed the model’s robustness, showing an AUC of 0.979, with a positive predictive value (PPV) of 0.908 and a negative predictive value (NPV) of 0.956 (**Figures 2B** and **S1B**). The external validation dataset, while showing an AUC of 0.959, provided valuable insights into the model’s generalizability across diverse populations (**Figures 2C** and **S1C**). Overall, the median cancer scores significantly differentiated between benign and malignant samples across all datasets (**Figures 2D, E**), underscoring the model’s potential for non-invasive CRC detection.

### Performance of CRC samples across different cancer stages

**Figure 3:**
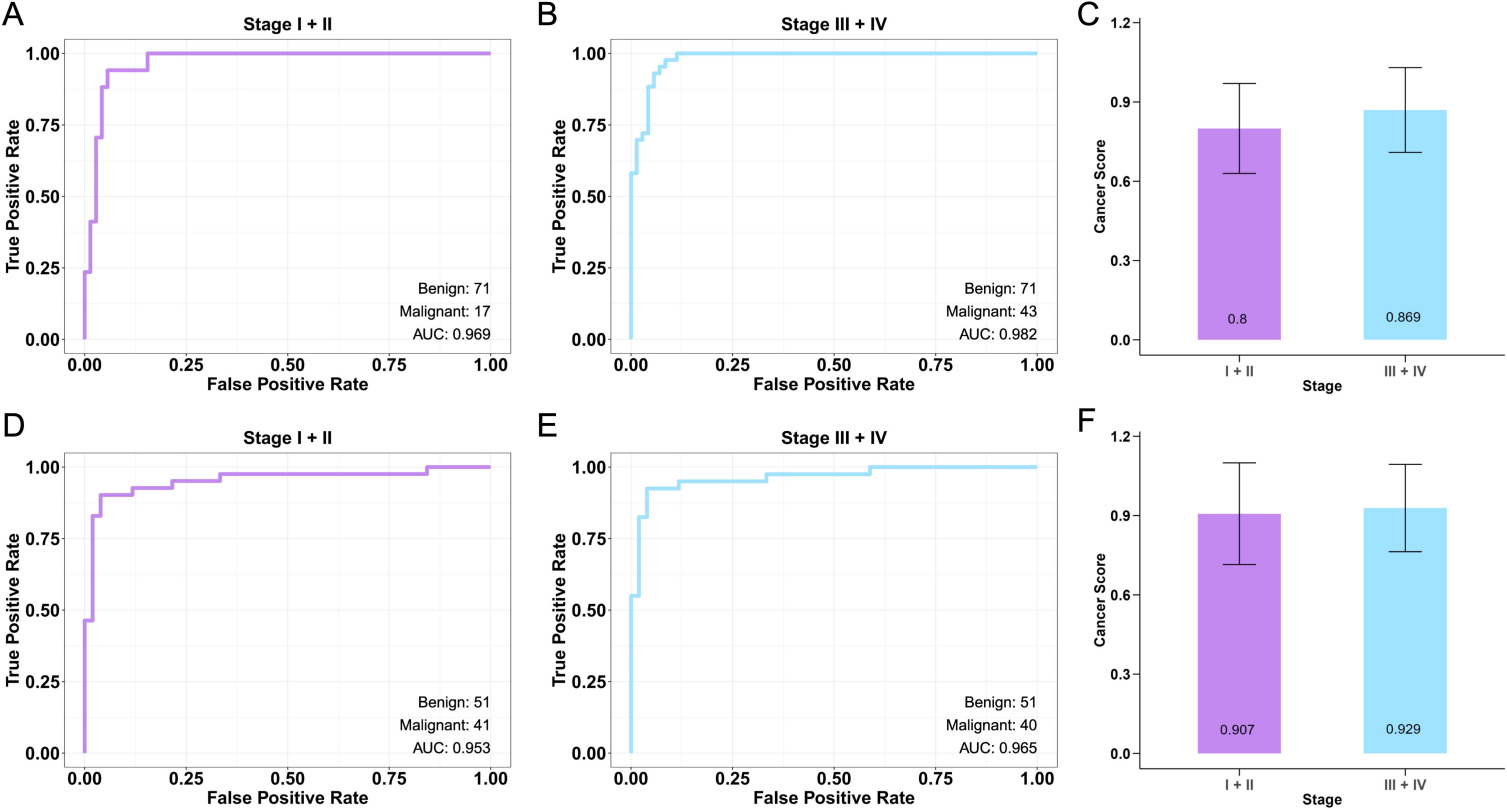
Performance of CRC cfDNA machine learning algorithm model across cancer stages. **(A)** The ROC curve for early stage (stage I + II) CRC in the internal validation dataset demonstrates the model’s ability to distinguish between benign and malignant samples. **(B)** ROC Curve for advanced stage (stage III + IV) CRC in the internal validation dataset. **(C)** Box plots comparing cancer scores between early stage and advanced stage CRC in the internal validation dataset. **(D)** The ROC curve for early stage CRC in the external validation dataset. **(E)** ROC Curve for advanced stage CRC in the external validation dataset. **(F)** Box plots comparing cancer scores between early stage and advanced stage in the external validation dataset.

The CRC cfDNA machine learning algorithm demonstrated strong performance in early stage (Stage I + II) CRC within the internal validation dataset, achieving a high AUC of 0.969 (**Figure 3A**). For advanced stage (Stage III + IV) CRC, the model maintained robust performance, with an AUC of 0.982 in the internal validation dataset (**Figure 3B**). Cancer scores were slightly higher in advanced stage cases, reflecting the model’s slightly better ability to detect advanced cancer (**Figure 3C**, P = 0.023). In the external validation dataset for early stage CRC, the model obtained an AUC of 0.953 (**Figure 3D**), and AUC of 0.965 in the advanced stage (**Figure 3E**). Furthermore, the cancer scores were slightly higher in advanced stage cases, consistent with the increased difficulty of detecting early stage cancer (**Figure 3F**, P = 0.277). The confusion matrices further highlight the model’s effectiveness in accurately classifying both benign and malignant samples, particularly in advanced stages. However, further refinement is needed to enhance early-stage detection (**Supplementary Figures S2A-D**). Overall, the results suggest a slightly difficulty in detecting early-stage CRC, as reflected by the lower cancer scores in these cases compared to those in advanced stages.

### Gender and age-based analysis of CRC cfDNA model performance

**Figure 4:**
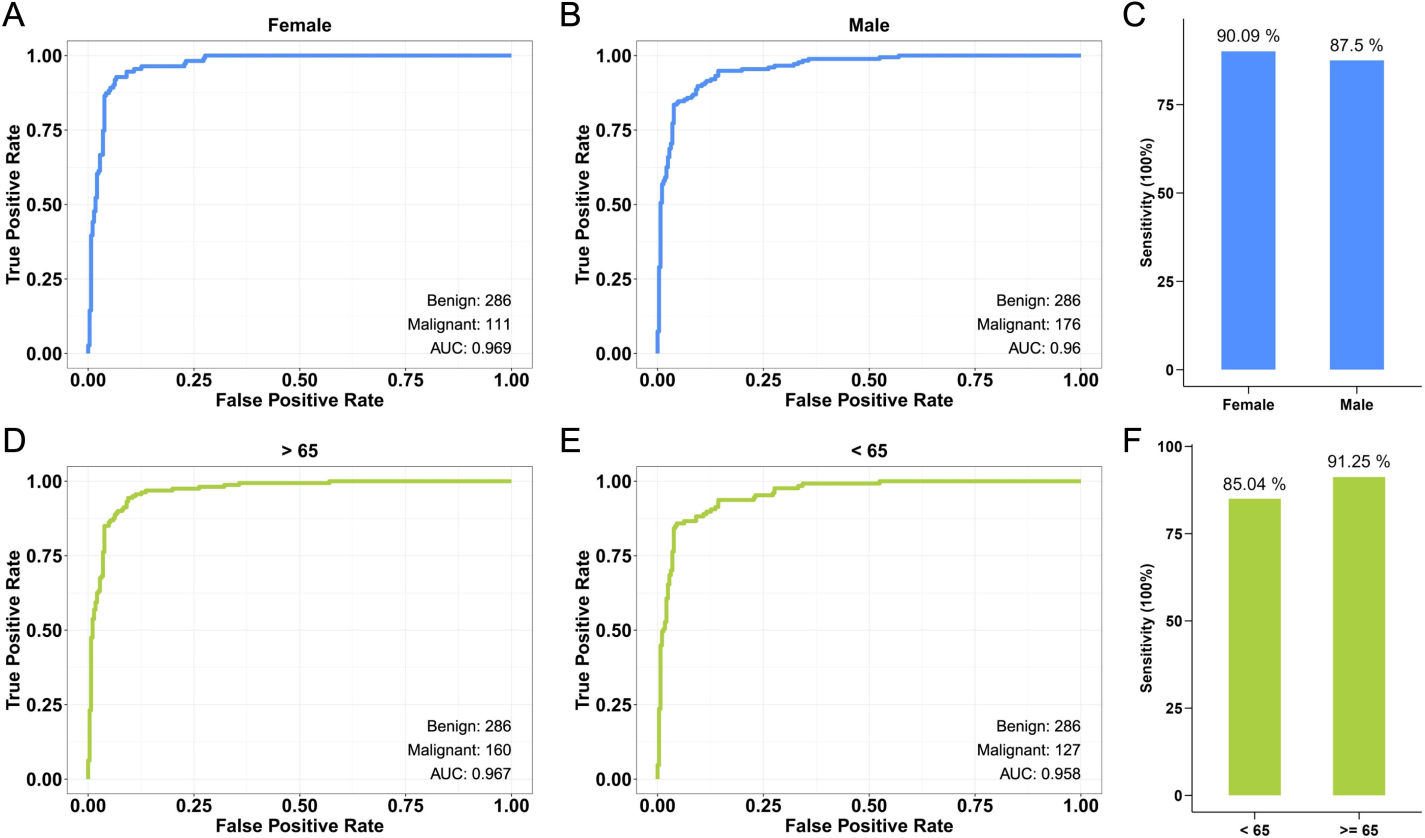
Performance of CRC cfDNA machine learning algorithm by gender and age groups. **(A)** The ROC curve shows the model’s performance in classifying CRC in female participants. **(B)** The ROC curve for male participants demonstrates the model’s classification performance. **(C)** Barplot of the sensitivity of the model between female and male participants. **(D)** The ROC curve illustrates the model’s performance in participants under 65 years old. **(E)** The ROC curve for participants aged 65 years and older. **(F)** Barplot of the model’s sensitivity in participants younger than 65 years and those 65 years or older.

The CRC cfDNA machine learning algorithm exhibited different performance levels when stratified by gender and age. For female participants, the model achieved a higher AUC of 0.969, compared to an AUC of 0.96 for male participants (**Figures 4A, B**). The sensitivity was also higher in females (90.1%) compared to males (87.5%) (**Figure 4C**). When analyzed by age, participants under 65 years old had a higher AUC of 0.967 compared to those 65 years or older, who had an AUC of 0.958 (**Figures 4D, E**). Sensitivity was similarly higher in the older age group (91.2%) than in the younger group (85.0%) (**Figure 4F**). These findings suggest that the model is applicable across different demographic groups, with notably better performance observed in females and younger participants.

### Differential analysis of cfDNA features in benign and malignant CRC samples

**Figure 5:**
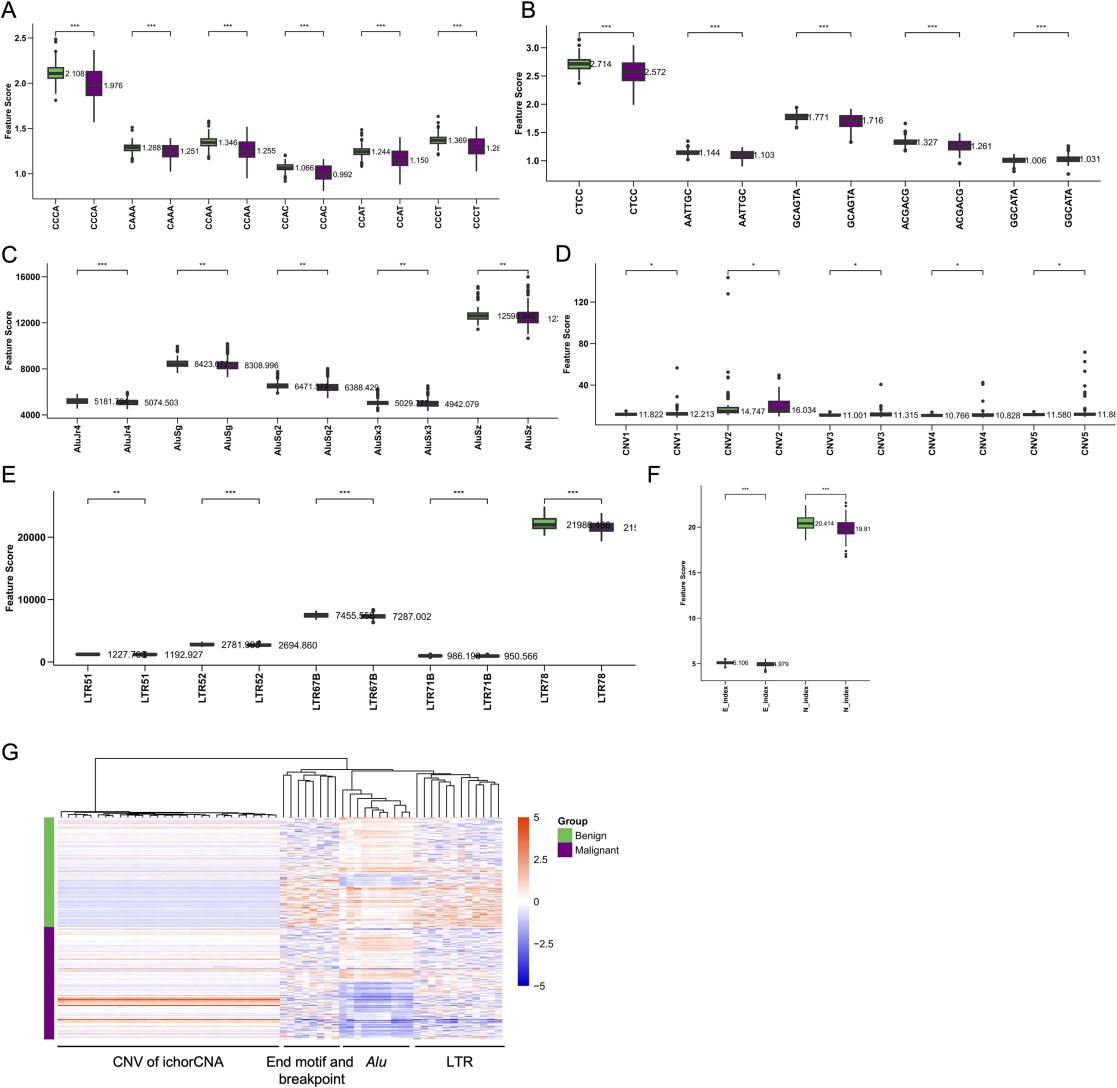
Differential analysis of cfDNA features in benign and malignant CRC samples. **(A)** Box plots showing the frequency distribution of various end End motifs between benign and malignant CRC samples. **(B)** Box plots illustrating the frequency distribution of different breakpoint motifs between benign and malignant samples. **(C)** Box plots comparing the frequencies of different *Alu* subfamilies (*AluY*, *AluS*, and *AluJ*) between benign and malignant samples. **(D)** Box plots depicting the frequency distribution of CNV from ichorCNA. **(E)** Box plots depicting the frequency distribution of various LTR subfamilies between benign and malignant samples. **(E)** Box plots comparing the frequencies of different E-index and N-index. **(G)** A heatmap displaying the top 30 cfDNA features that most significantly differentiate benign from malignant CRC samples (CNV of ichorCNA, End motif and breakpoint, *Alu* and LTR).

The differential analysis of cfDNA features between benign and CRC samples revealed significant and nuanced variations across key motifs, elements, and repeats, underscoring the complexity of colorectal cancer progression. End motifs such as CCCA, CAAA, CCAA, CCAC, CCAT and CCCT were notably more frequent in malignant samples, suggesting their potential role in tumor development, whereas motifs like CAAA and CCAC exhibited a slight decrease in frequency in malignant samples, which may indicate selective motif preservation or loss during cancer progression (**Figure 5A**). Breakpoint motifs also displayed significant differences, with malignant samples showing elevated frequencies of motifs such as CTCC and AATTGC, while motifs like GCAGTA, ACGACG, and GGCATA were less frequent, possibly reflecting the distinct genomic instability in cancerous cells (**Figure 5B**). The analysis of *Alu* elements revealed that benign samples generally exhibited higher frequencies across various *Alu* subfamilies, including *AluY*, *AluS*, and *AluJ*, compared to malignant samples, highlighting the potential protective role or differential regulation of these elements in non-cancerous tissues (**Figure 5C**). Similarly, CNV features derived from ichorCNA showed only subtle differences between malignant and benign samples, with no prominent large-scale copy number alterations observed. These weak but detectable CNV signals were more frequently present in malignant CRC samples than in benign controls (**Figure 5D**). The frequencies of RNA elements such as LTRs also differed between the groups, with malignant samples showing higher levels of these elements, suggesting that these RNA components may contribute to or result from the altered transcriptional landscape in cancer (**Figure 5E**). Additional, significant differences in the E-index and N-index were observed between malignant and benign samples, with malignant samples exhibiting distinct fragmentation-associated nucleotide bias patterns compared with benign controls (**Figure 5F**). Finally, the heatmap of the top 30 differential cfDNA features clearly distinguished between benign and malignant samples, pinpointing specific features that could serve as robust biomarkers for CRC detection and offering insights into the molecular underpinnings of colorectal malignancy (**Figure 5G**). These findings collectively underscore the rich potential of cfDNA fragmentomic analysis as a powerful tool for distinguishing benign from malignant colorectal conditions and for advancing the understanding of CRC biology.

In addition, the Griffin method was employed to analyze cfDNA coverage amplitudes in benign and malignant CRC samples, revealing distinct patterns that reflect variations in chromatin accessibility and nucleosome positioning (**Supplementary Figures S3A, B**, *P* < 0.001). The LIQUORICE algorithm further elucidated these differences by generating area over the curve (AOC) metrics for cfDNA depth, which displayed broader and more dispersed distributions in malignant samples, suggesting altered fragmentation profiles associated with cancer (**Supplementary Figures S3C, D**, *P* < 0.001). The analysis of DNA transposons offered additional insights into the differential regulation of these elements in colorectal cancer. The DNA_hAT_Charlie and DNA_hAT_Tip100 families of DNA transposons exhibited significantly lower frequencies in malignant samples compared to benign ones, indicating possible dysregulation or suppression of these transposons in the cancerous state (**Supplementary Figure S3E**). Furthermore, the frequency analysis of other DNA transposons (DNA_TcMar-Tigger), including Tigger15a, Tigger19a, and Tigger19b, revealed a complex pattern of transposon activity. These elements were generally more active in benign samples than in malignant ones, reflecting the intricate dynamics of transposon regulation during colorectal cancer development (**Supplementary Figure S3F**). These findings underscore the potential of using advanced computational algorithms and transposon profiling to uncover the molecular mechanisms underlying colorectal cancer and to effectively distinguish between benign and malignant conditions.

## Discussion

In this study, we developed and validated a machine learning algorithm based on cfDNA fragmentomics for the non-invasive early detection of CRC. During feature extraction, we incorporated a wide range of features, including fragment length, 3′ end motifs, N-index, E-index, CNV features derived from ichorCNA, Griffin-based features, LIQUORICE-based features, as well as breakpoint motifs, *Alu* elements, LTRs, RNA elements, and transposable elements, comprehensively covering various structural and sequence-level characteristics of cfDNA. Compared with previous studies that typically focused only on breakpoints and 3′ end motifs, our feature set is more comprehensive and multidimensional, enabling a deeper exploration of tumor-associated cfDNA fragmentomic signatures and potentially improving detection sensitivity and specificity. In particular, the inclusion of the N-index and E-index enables quantitative characterization of cfDNA fragmentation patterns by capturing nucleotide composition biases at fragment ends, which have been shown to reflect tumor-associated enzymatic cleavage preferences. CNV features derived from ichorCNA provide genome-wide information on somatic copy number alterations, a hallmark of chromosomal instability in colorectal cancer, thereby complementing sequence-based fragmentomic signals. Griffin-based features further quantify regional fragmentation patterns across the genome, facilitating the detection of cancer-specific alterations in cfDNA coverage and fragmentation profiles. In parallel, LIQUORICE-based features characterize local fragmentation behaviors around functional genomic elements, allowing the identification of tumor-related deviations in nucleosome positioning and chromatin accessibility. Together, these features capture complementary aspects of cfDNA biology at both global and local levels, enhancing the model’s ability to robustly discriminate CRC from benign conditions. The results demonstrated that our model achieved strong robustness and accuracy across multiple datasets, with particularly notable performance in advanced-stage CRC patients, females, and individuals across different age groups. This study underscores the great potential of cfDNA fragmentomic analysis in non-invasive CRC detection.

Our results demonstrate that CRC prediction based on cfDNA fragmentation patterns achieves high sensitivity and specificity, with an AUC of 0.979, which is consistent with the findings reported by Cao et al. (29) and Zhou et al. (30). Moreover, our model exhibits robust predictive performance for both early-stage (I+II) and advanced-stage (III+IV) colorectal cancers. The predictive capability of the same model may vary across different patient subgroups stratified by clinical characteristics, including tumor stage, sex, age, and tumor size. Generally, the model shows higher sensitivity for advanced-stage tumors compared to early-stage tumors (38). In the study by Cao et al. (29), at a specific cutoff, the model demonstrated improved sensitivity for tumors larger than 3 cm. In addition, our findings indicate that the model performs better in elderly patients, which may be attributed to age-related differences in cfDNA fragmentation patterns or the tendency of older patients to present with more advanced tumor stages.

One of the key findings of this study is the differential behavior of *Alu* elements between benign and CRC samples. *Alu* elements, which are short interspersed nuclear elements (SINEs), are known to be highly repetitive sequences in the human genome and are involved in various genomic processes, including gene regulation, genome evolution, and genomic instability (21, 22). Our analysis revealed that benign samples generally exhibited higher frequencies of various *Alu* subfamilies, such as *AluY*, *AluS*, and *AluJ*, compared to malignant samples. This suggests that *Alu* elements may play a protective role in maintaining genomic integrity in non-cancerous tissues. Conversely, the lower frequency of *Alu* elements in malignant samples may reflect their potential dysregulation during cancer progression, contributing to genomic instability and the development of CRC.

The role of other repetitive elements, such as LTRs and transposable elements, was also highlighted in our analysis. LTR subfamilies like LTR_ERVL and LTR_Gypsy were more prevalent in malignant samples, indicating their possible involvement in retrotransposon activation and genomic instability in cancer. The differential regulation of DNA transposons, including DNA_hAT_Charlie and DNA_hAT_Tip100, further underscores the complex dynamics of transposon activity in CRC. These findings suggest that repetitive elements, including *Alu* sequences, LTRs, and DNA transposons, could serve as valuable biomarkers for CRC detection and provide insights into the underlying mechanisms of colorectal malignancy.

Furthermore, the features corresponding to ARTEMIS and ARTEMIS-DELFI have indeed been incorporated into our input features for model training and validation. The novelty of the ARTEMIS-DELFI approach lies not in the initial observation of alterations in repetitive elements but in its paradigm shift from a hypothesis-driven investigation targeting specific elements to an unbiased, genome-wide systematic discovery. The earlier study by Douville et al. utilized a targeted amplification technique (RealSeqS) focused specifically on *Alu* elements, identifying a global reduction in the *AluS* subfamily within the cell-free DNA (cfDNA) of cancer patients (22). In contrast, the ARTEMIS method developed by Annapragada et al. employs a fundamentally new, alignment-free kmer analysis strategy to conduct a systematic scan of the entire repeatome, encompassing 1,280 distinct types of repetitive elements across six major families, including SINEs, LINEs, LTRs, and Satellite DNA, from standard whole-genome sequencing data (21). This comprehensive, untargeted methodology enabled the discovery of 820 repetitive elements previously unknown to be altered in cancer, significantly expanding the catalog of genomic changes associated with tumorigenesis beyond the specific *AluS* finding (21). This foundational innovation allows ARTEMIS to link these widespread repeat landscape alterations to deeper mechanisms of cancer genomic instability, such as chromosomal structural variations and epigenetic states. Furthermore, the integration of the ARTEMIS score with fragmentomic features (DELFI) not only enhanced the detection performance for early-stage lung and liver cancer in independent validation cohorts (achieving an AUC of up to 0.91) but also extended its clinical utility to novel dimensions including prognosis assessment, therapy response monitoring, and determining the tissue of origin for tumors (39). The value of this combined approach thus extends far beyond mere cancer detection, offering a more powerful and versatile framework for cancer genomics and liquid biopsy applications.

However, several limitations must be acknowledged. First, while the model demonstrated high accuracy in the training and internal validation datasets, its performance in the external validation dataset was lower, particularly in early-stage CRC. This suggests that the model may require further refinement to enhance its generalizability across diverse populations and early-stage detection. Additionally, although we employed advanced machine learning algorithms GLM, the model’s reliance on specific genomic features may limit its applicability to other cancer types or broader clinical settings.

Another limitation is the need for a larger and more diverse cohort to validate the model’s effectiveness across different populations and ethnicities. The study was conducted using samples from a single institution, and the inclusion of participants from multiple centers and diverse backgrounds would strengthen the generalizability of the findings. Moreover, the computational complexity of the machine learning algorithms used in this study may pose challenges for routine clinical implementation, necessitating the development of more streamlined and accessible models.

Despite these limitations, the findings of this study offer promising avenues for the clinical application of cfDNA fragmentomic analysis in CRC detection. The incorporation of *Alu* elements, LTRs, and other repetitive sequences into predictive models provides a novel approach to identifying genomic signatures associated with colorectal cancer. Future research should focus on refining these models, expanding the cohort size, and exploring the role of repetitive elements in other cancer types. Additionally, integrating cfDNA analysis with other diagnostic modalities, such as imaging or proteomics, could enhance the overall accuracy and reliability of CRC detection.

## Conclusions

In conclusion, this study demonstrates that cfDNA fragmentomic analysis represents a promising approach for the non-invasive detection of colorectal cancer. By integrating a comprehensive set of fragmentomic features, including *Alu* and other repetitive elements, our machine learning model achieved robust performance across both internal and strictly independent external validation cohorts. Although the results indicate strong discriminatory ability, further large-scale, prospective validation and methodological refinement are warranted before clinical translation. Nonetheless, these findings support the potential utility of advanced computational frameworks and transposon-informed fragmentomic profiling in enhancing non-invasive cancer diagnostics and improving our understanding of the molecular alterations associated with colorectal carcinogenesis.

## Data Availability

The datasets presented in this study can be found in online repositories. The names of the repository/repositories and accession number(s) can be found in the article/**Supplementary Material**.

## Supplementary material

Supplementary Figure 1 Confusion matrices for CRC cfDNA machine learning algorithm model (GLM). **(A)** The confusion matrix displays the classification performance of the CRC cfDNA machine learning algorithm on the training dataset. **(B)** This confusion matrix represents the model’s performance on the internal validation dataset. Of the 37 benign samples. **(C)** The confusion matrix for the external validation dataset.

Supplementary Figure 2 Confusion matrices for CRC cfDNA machine learning algorithm across cancer stages. **(A)** The confusion matrix for early stage CRC in the internal validation dataset. **(B)** Confusion matrix for advanced stage CRC in the internal validation dataset. **(C)** Confusion matrix for stage I + II in external validation dataset. **(D)** Confusion matrix for stage III + IV in external validation dataset.

Supplementary Figure 3 Analysis of cfDNA features in benign and malignant CRC samples using different algorithms and DNA transposons. **(A, B)** The Griffin method was employed to analyze cfDNA coverage amplitudes across benign and malignant CRC samples. **(C, D)**. The LIQUORICE algorithm was used to generate contour plots showing the area over the curve (AOC) for total cfDNA depth across benign and malignant samples. **(E)** Box plots comparing the frequencies of the hAT-Charlie and hAT-Tip100 families of DNA transposons between benign and malignant samples. **(F)** Box plots showing the frequency distribution of other DNA transposons (DNA_TcMar-Tigger) between benign and malignant samples.

Supplementary Table 1 Clinical information of samples.

Supplementary Table 2 Sensitivity and specificity at fixed specificity thresholds.

## References

[1] Keum N Giovannucci E . Global burden of colorectal cancer: emerging trends, risk factors and prevention strategies. Nat Rev Gastroenterol Hepatol. (2019) 16:713–32. doi: 10.1038/s41575-019-0189-8, PMID: 31455888

[2] Li N Lu B Luo C Cai J Lu M Zhang Y . Incidence, mortality, survival, risk factor and screening of colorectal cancer: A comparison among China, Europe, and northern America. Cancer Lett. (2021) 522:255–68. doi: 10.1016/j.canlet.2021.09.034, PMID: 34563640

[3] Murphy CC Zaki TA . Changing epidemiology of colorectal cancer - birth cohort effects and emerging risk factors. Nat Rev Gastroenterol Hepatol. (2024) 21:25–34. doi: 10.1038/s41575-023-00841-9, PMID: 37723270

[4] Scully A Cheung I . Colorectal cancer screening: fecal occult blood test literature review for occupational health nurses. Workplace Health Saf. (2016) 64:114–22; quiz 123. doi: 10.1177/2165079915616647, PMID: 26941081

[5] Beniwal SS Lamo P Kaushik A Lorenzo-Villegas DL Liu Y MohanaSundaram A . Current status and emerging trends in colorectal cancer screening and diagnostics. Biosensors. (2023) 13:926. doi: 10.3390/bios13100926, PMID: 37887119 PMC10605407

[6] Chen X Gole J Gore A He Q Lu M Min J . Non-invasive early detection of cancer four years before conventional diagnosis using a blood test. Nat Commun. (2020) 11:3475. doi: 10.1038/s41467-020-17316-z, PMID: 32694610 PMC7374162

[7] Tang L Chang SJ Chen C-J Liu J-T . Non-invasive blood glucose monitoring technology: A review. Sensors. (2020) 20:6925. doi: 10.3390/s20236925, PMID: 33291519 PMC7731259

[8] Fitzgerald RC Antoniou AC Fruk L Rosenfeld N . The future of early cancer detection. Nat Med. (2022) 28:666–77. doi: 10.1038/s41591-022-01746-x, PMID: 35440720

[9] Kumar P Gupta S Das BC . Saliva as a potential non-invasive liquid biopsy for early and easy diagnosis/prognosis of head and neck cancer. Transl Oncol. (2024) 40:101827. doi: 10.1016/j.tranon.2023.101827, PMID: 38042138 PMC10701368

[10] Qi T Pan M Shi H Wang L Bai Y Ge Q . Cell-free DNA fragmentomics: the novel promising biomarker. Int J Mol Sci. (2023) 24:1503. doi: 10.3390/ijms24021503, PMID: 36675018 PMC9866579

[11] Hu Y Zhao Y Zhang Y Chen W Zhang H Jin X . Cell-free DNA: a promising biomarker in infectious diseases. Trends Microbiol. (2025) 33:421–33. doi: 10.1016/j.tim.2024.06.005, PMID: 38997867

[12] Tivey A Church M Rothwell D Dive C Cook N . Circulating tumour DNA - looking beyond the blood. Nat Rev Clin Oncol. (2022) 19:600–12. doi: 10.1038/s41571-022-00660-y, PMID: 35915225 PMC9341152

[13] Pomerantz T Brooks R . Circulating tumor DNA (ctDNA) and its role in gynecologic Malignancies. Curr Treat Options Oncol. (2024) 25:510–22. doi: 10.1007/s11864-024-01180-w, PMID: 38472567

[14] Liu Y . At the dawn: cell-free DNA fragmentomics and gene regulation. Br J Cancer. (2022) 126:379–90. doi: 10.1038/s41416-021-01635-z, PMID: 34815523 PMC8810841

[15] Jiang P Lo YMD . Enhanced cancer detection from cell-free DNA. Nat Biotechnol. (2022) 40:473–4. doi: 10.1038/s41587-021-01207-9, PMID: 35361997

[16] Guo W Chen X Liu R Liang N Ma Q Bao H . Sensitive detection of stage I lung adenocarcinoma using plasma cell-free DNA breakpoint motif profiling. EBioMedicine. (2022) 81:104131. doi: 10.1016/j.ebiom.2022.104131, PMID: 35780566 PMC9251329

[17] Esfahani MS Hamilton EG Mehrmohamadi M Nabet BY Alig SK King DA . Inferring gene expression from cell-free DNA fragmentation profiles. Nat Biotechnol. (2022) 40:585–97. doi: 10.1038/s41587-022-01222-4, PMID: 35361996 PMC9337986

[18] Qin X Bai Y Zhou S Shi H Liu X Wang S . Early diagnosis of brain metastases using cerebrospinal fluid cell-free DNA-based breakpoint motif and mutational features in lung cancer. Clin Transl Med. (2023) 13:e1221. doi: 10.1002/ctm2.1221, PMID: 36929639 PMC10019768

[19] Serpas L Chan RWY Jiang P Ni M Sun K Rashidfarrokhi A . Dnase1l3 deletion causes aberrations in length and end-motif frequencies in plasma DNA. Proc Natl Acad Sci U S A. (2019) 116:641–9. doi: 10.1073/pnas.1815031116, PMID: 30593563 PMC6329986

[20] Jiang P Sun K Peng W Cheng SH Ni M Yeung PC . Plasma DNA end-motif profiling as a fragmentomic marker in cancer, pregnancy, and transplantation. Cancer Discov. (2020) 10:664–73. doi: 10.1158/2159-8290.CD-19-0622, PMID: 32111602

[21] Annapragada AV Niknafs N White JR Bruhm DC Cherry C Medina JE . Genome-wide repeat landscapes in cancer and cell-free DNA. Sci Transl Med. (2024) 16:eadj9283. doi: 10.1126/scitranslmed.adj9283, PMID: 38478628 PMC11323656

[22] Douville C Lahouel K Kuo A Grant H Avigdor BE Curtis SD . Machine learning to detect the SINEs of cancer. Sci Transl Med. (2024) 16:eadi3883. doi: 10.1126/scitranslmed.adi3883, PMID: 38266106 PMC11210392

[23] Hoyt SJ Storer JM Hartley GA Grady PGS Gershman A de Lima LG . From telomere to telomere: The transcriptional and epigenetic state of human repeat elements. Science. (2022) 376:eabk3112. doi: 10.1126/science.abk3112, PMID: 35357925 PMC9301658

[24] Stanley KE Jatsenko T Tuveri S Sudhakaran D Lannoo L Van Calsteren K . Cell type signatures in cell-free DNA fragmentation profiles reveal disease biology. Nat Commun. (2024) 15:2220. doi: 10.1038/s41467-024-46435-0, PMID: 38472221 PMC10933257

[25] Babaian A Mager DL . Endogenous retroviral promoter exaptation in human cancer. Mob DNA. (2016) 7:24. doi: 10.1186/s13100-016-0080-x, PMID: 27980689 PMC5134097

[26] Zhou X Cheng Z Dong M Liu Q Yang W Liu M . Tumor fractions deciphered from circulating cell-free DNA methylation for cancer early diagnosis. Nat Commun. (2022) 13:7694. doi: 10.1038/s41467-022-35320-3, PMID: 36509772 PMC9744803

[27] Zhang K Fu R Liu R Su Z . Circulating cell-free DNA-based multi-cancer early detection. Trends Cancer. (2024) 10:161–74. doi: 10.1016/j.trecan.2023.08.010, PMID: 37709615

[28] Ma X Chen Y Tang W Bao H Mo S Liu R . Multi-dimensional fragmentomic assay for ultrasensitive early detection of colorectal advanced adenoma and adenocarcinoma. J Hematol OncolJ Hematol Oncol. (2021) 14:175. doi: 10.1186/s13045-021-01189-w, PMID: 34702327 PMC8549237

[29] Cao Y Wang N Wu X Tang W Bao H Si C . Multidimensional fragmentomics enables early and accurate detection of colorectal cancer. Cancer Res. (2024) 84:3286–95. doi: 10.1158/0008-5472.CAN-23-3486, PMID: 39073362

[30] Zhou J Pan Y Wang S Wang G Gu C Zhu J . Early detection and stratification of colorectal cancer using plasma cell-free DNA fragmentomic profiling. Genomics. (2024) 116:110876. doi: 10.1016/j.ygeno.2024.110876, PMID: 38849019

[31] Budhraja KK McDonald BR Stephens MD Contente-Cuomo T Markus H Farooq M . Genome-wide analysis of aberrant position and sequence of plasma DNA fragment ends in patients with cancer. Sci Transl Med. (2023) 15:eabm6863. doi: 10.1126/scitranslmed.abm6863, PMID: 36630480 PMC10080578

[32] Adalsteinsson VA Ha G Freeman SS Choudhury AD Stover DG Parsons HA . Scalable whole-exome sequencing of cell-free DNA reveals high concordance with metastatic tumors. Nat Commun. (2017) 8:1324. doi: 10.1038/s41467-017-00965-y, PMID: 29109393 PMC5673918

[33] An Y Zhao X Zhang Z Xia Z Yang M Ma L . DNA methylation analysis explores the molecular basis of plasma cell-free DNA fragmentation. Nat Commun. (2023) 14:287. doi: 10.1038/s41467-023-35959-6, PMID: 36653380 PMC9849216

[34] Ju J Zhao X An Y Yang M Zhang Z Liu X . Cell-free DNA end characteristics enable accurate and sensitive cancer diagnosis. Cell Rep Methods. (2024) 4:100877. doi: 10.1016/j.crmeth.2024.100877, PMID: 39406232 PMC11573786

[35] Van Roy N van der Linden M Menten B Dheedene A Vandeputte C Van Dorpe J . Shallow whole genome sequencing on circulating cell-free DNA allows reliable noninvasive copy-number profiling in neuroblastoma patients. Clin Cancer Res Off J Am Assoc Cancer Res. (2017) 23:6305–14. doi: 10.1158/1078-0432.CCR-17-0675, PMID: 28710315

[36] Doebley A-L Ko M Liao H Cruikshank AE Santos K Kikawa C . A framework for clinical cancer subtyping from nucleosome profiling of cell-free DNA. Nat Commun. (2022) 13:7475. doi: 10.1038/s41467-022-35076-w, PMID: 36463275 PMC9719521

[37] Hiatt JB Doebley A-L Arnold HU Adil M Sandborg H Persse TW . Molecular phenotyping of small cell lung cancer using targeted cfDNA profiling of transcriptional regulatory regions. Sci Adv. (2024) 10:eadk2082. doi: 10.1126/sciadv.adk2082, PMID: 38598634 PMC11006233

[38] Chung DC Gray DM Singh H Issaka RB Raymond VM Eagle C . A cell-free DNA blood-based test for colorectal cancer screening. N Engl J Med. (2024) 390:973–83. doi: 10.1056/NEJMoa2304714, PMID: 38477985

[39] Hruban C Bruhm DC Chen IM Koul S Annapragada AV Vulpescu NA . Genome-wide analyses of cell-free DNA for therapeutic monitoring of patients with pancreatic cancer. Sci Adv. (2025) 11:eads5002. doi: 10.1126/sciadv.ads5002, PMID: 40397745 PMC12094228

