# Supplementary figures and images for: Leveraging cfDNA fragmentomic features for the early detection of colorectal cancer
Source: Front Immunol. 2026 Jan 28;17:1705156. doi: 10.3389/fimmu.2026.1705156 (PMC12891135; doi:10.3389/fimmu.2026.1705156)

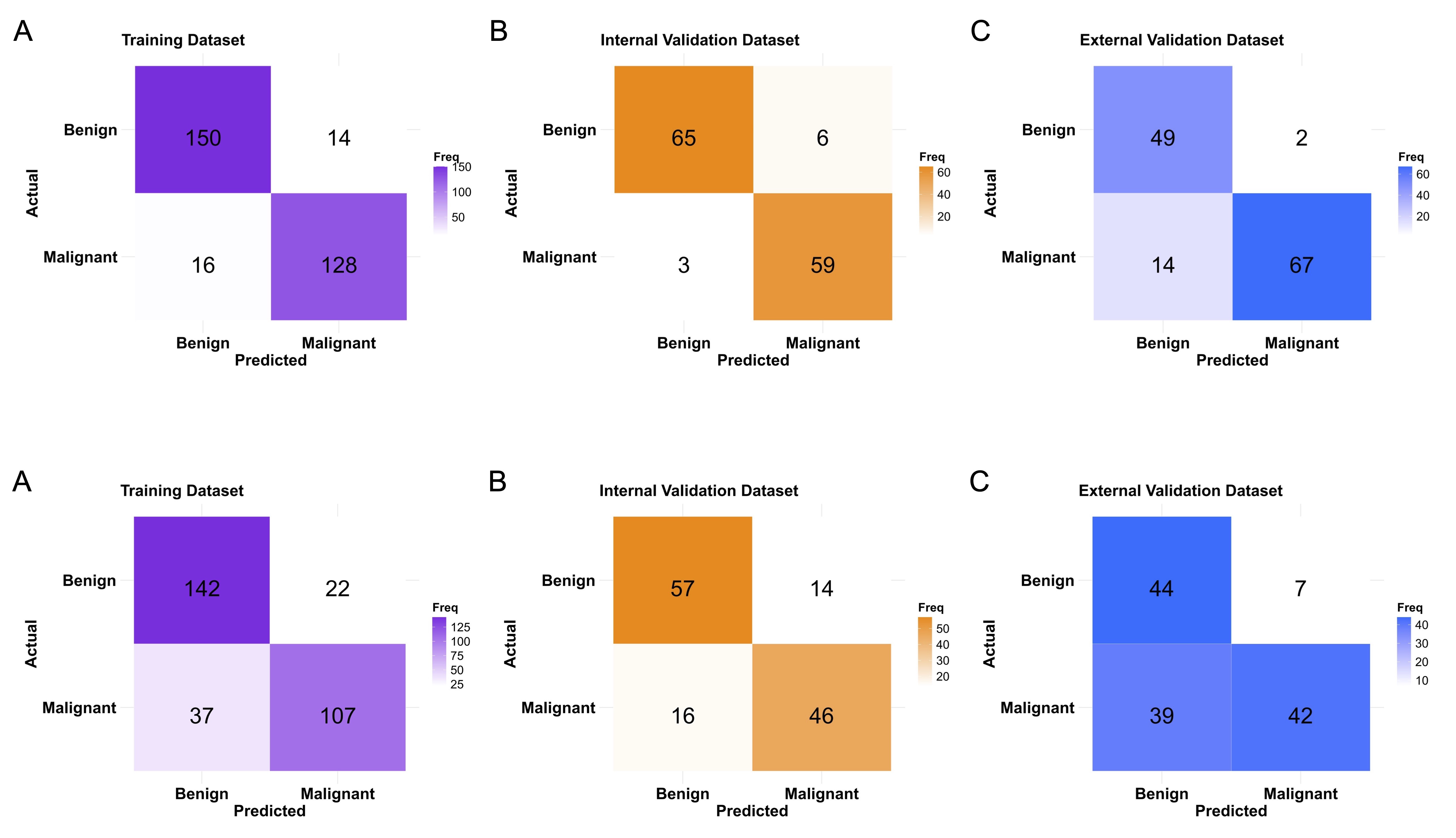

Supplement: Supplementary Figure 1 — Confusion matrices for CRC cfDNA machine learning algorithm model (GLM). (A) The confusion matrix displays the classification performance of the CRC cfDNA machine learning algorithm on the training dataset. (B) This confusion matrix represents the model’s performance on the internal validation dataset. Of the 37 benign samples. (C) The confusion matrix for the external validation dataset. [file Image1.jpeg]

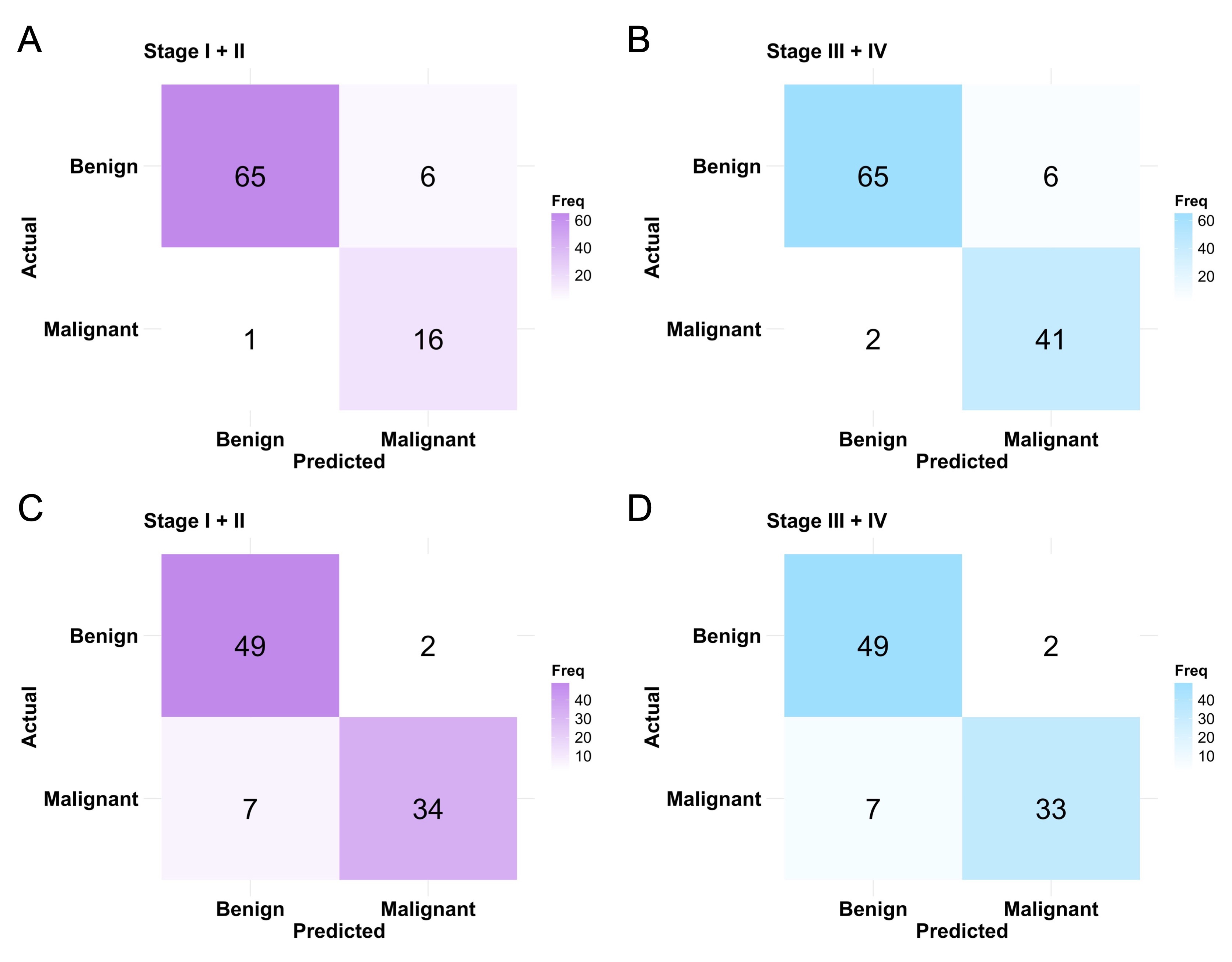

Supplement: Supplementary Figure 2 — Confusion matrices for CRC cfDNA machine learning algorithm across cancer stages. (A) The confusion matrix for early stage CRC in the internal validation dataset. (B) Confusion matrix for advanced stage CRC in the internal validation dataset. (C) Confusion matrix for stage I + II in external validation dataset. (D) Confusion matrix for stage III + IV in external validation dataset. [file Image2.jpeg]

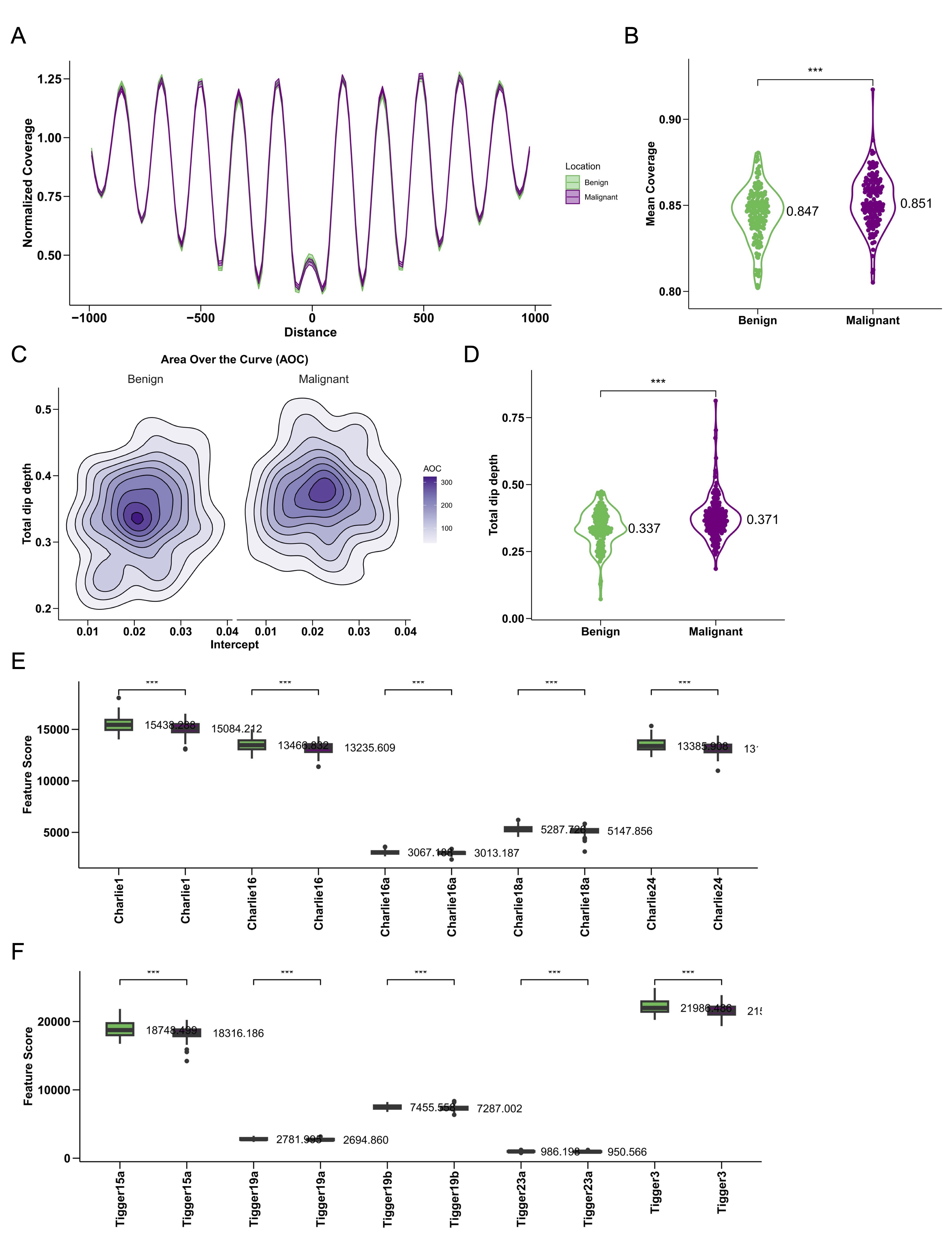

Supplement: Supplementary Figure 3 — Analysis of cfDNA features in benign and malignant CRC samples using different algorithms and DNA transposons. (A, B) The Griffin method was employed to analyze cfDNA coverage amplitudes across benign and malignant CRC samples. (C, D). The LIQUORICE algorithm was used to generate contour plots showing the area over the curve (AOC) for total cfDNA depth across benign and malignant samples. (E) Box plots comparing the frequencies of the hAT-Charlie and hAT-Tip100 families of DNA transposons between benign and malignant samples. (F) Box plots showing the frequency distribution of other DNA transposons (DNA_TcMar-Tigger) between benign and malignant samples. [file Image3.jpeg]
